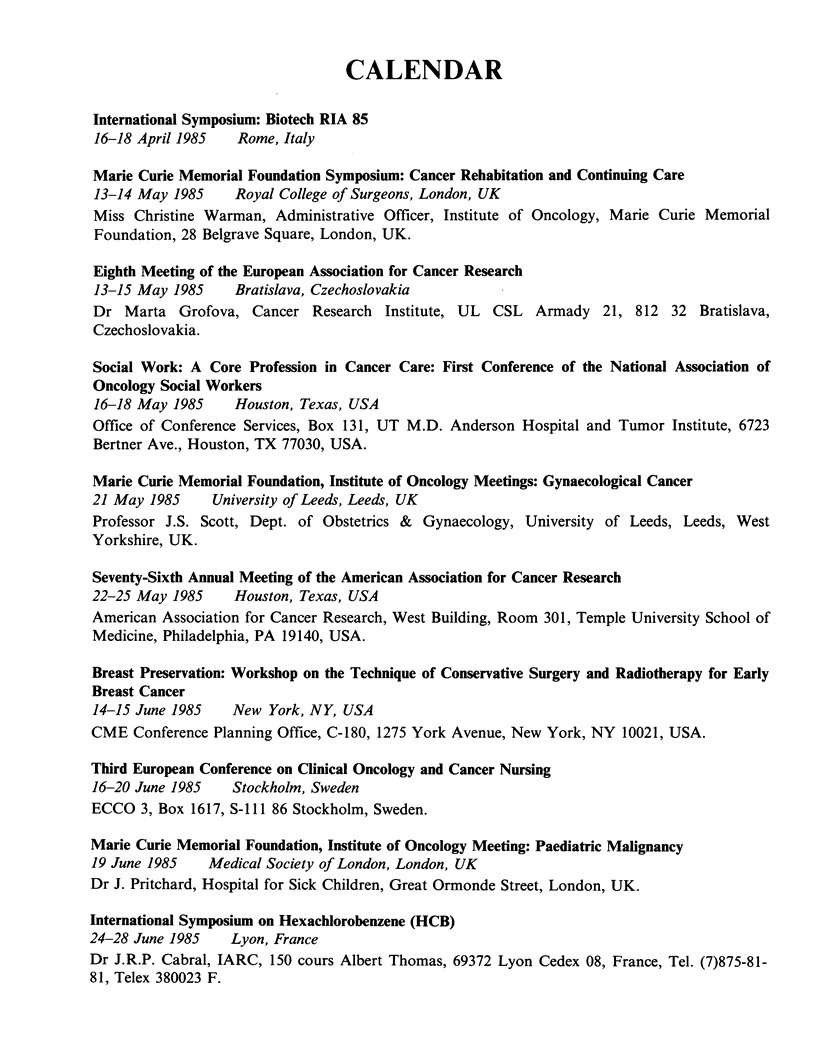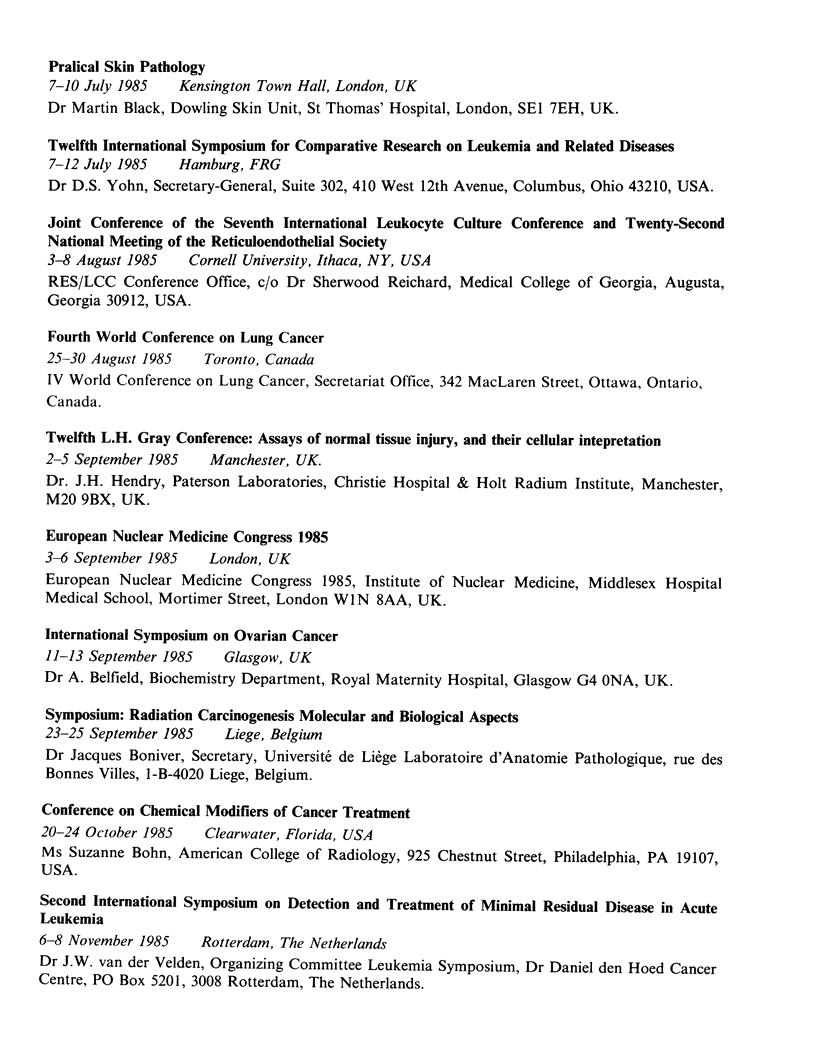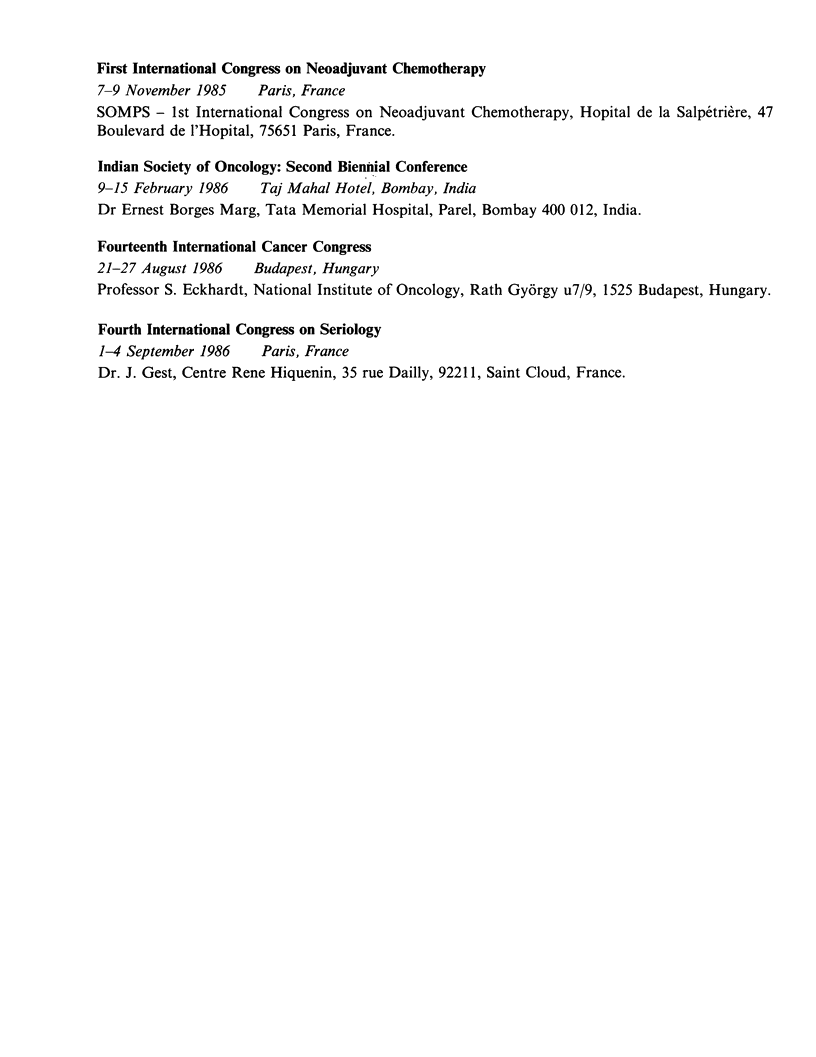# Calendar

**Published:** 1985-04

**Authors:** 


### CALENDAR

**International Symposium: Biotech RIA 85**
*16–18 April 1985 Rome, Italy*

**Marie Curie Memorial Foundation Symposium: Cancer Rehabilitation and Continuing Care**
*13–14 May 1985 Royal College of Surgeons, London, UK*
Miss Christine Warman, Administrative Officer, Institute of Oncology, Marie Curie Memorial Foundation, 28 Belgrave Square, London, UK.

**Eighth Meeting of the European Association for Cancer Research**
*13–15 May 1985 Bratislava, Czechoslovakia*
Dr Marta Grofova, Cancer Research Institute, UL CSL Armady 21, 812 32 Bratislava, Czechoslovakia.

**Social Work: A Core Profession in Cancer Care: First Conference of the National Association of Oncology Social Workers**
*16–18 May 1985 Houston, Texas, USA*
Office of Conference Services, Box 131, UT M.D. Anderson Hospital and Tumor Institute, 6723 Bertner Ave., Houston, TX 77030, USA.

**Marie Curie Memorial Foundation, Institute of Oncology Meetings: Gynaecological Cancer**
*21 May 1985 University of Leeds, Leeds, UK*
Professor J.S. Scott, Dept. of Obstetrics & Gynaecology, University of Leeds, Leeds, West Yorkshire, UK.

**Seventy-Sixth Annual Meeting of the American Association for Cancer Research**
*22–25 May 1985 Houston, Texas, USA*
American Association for Cancer Research, West Building, Room 301, Temple University School of Medicine, Philadelphia, PA 19140, USA.

**Breast Preservation: Workshop on the Technique of Conservative Surgery and Radiotherapy for Early Breast Cancer**
*14–15 June 1985 New York, NY, USA*
CME Conference Planning Office, C-180, 1275 York Avenue, New York, NY 10021, USA.

**Third European Conference on Clinical Oncology and Cancer Nursing**
*16–20 June 1985 Stockholm, Sweden*
ECCO 3, Box 1617, S-111 86 Stockholm, Sweden.

**Marie Curie Memorial Foundation, Institute of Oncology Meeting: Paediatric Malignancy**
*19 June 1985 Medical Society of London, London, UK*
Dr J. Pritchard, Hospital for Sick Children, Great Ormonde Street, London, UK.

**International Symposium on Hexachlorobenzene (HCB)**
*24–28 June 1985 Lyon, France*
Dr J.R.P. Cabral, IARC, 150 cours Albert Thomas, 69372 Lyon Cedex 08, France, Tel. (7)875-81-81, Telex 380023 F.

**Pralical Skin Pathology**
*7–10 July 1985*    *Kensington Town Hall, London, UK*
Dr Martin Black, Dowling Skin Unit, St Thomas' Hospital, London, SE1 7EH, UK.

**Twelfth International Symposium for Comparative Research on Leukemia and Related Diseases**
*7–12 July 1985*    *Hamburg, FRG*
Dr D.S. Yohn, Secretary-General, Suite 302, 410 West 12th Avenue, Columbus, Ohio 43210, USA.

**Joint Conference of the Seventh International Leukocyte Culture Conference and Twenty-Second National Meeting of the Reticuloendothelial Society**
*3–8 August 1985*    *Cornell University, Ithaca, NY, USA*
RES/LCC Conference Office, c/o Dr Sherwood Reichard, Medical College of Georgia, Augusta, Georgia 30912, USA.

**Fourth World Conference on Lung Cancer**
*25–30 August 1985*    *Toronto, Canada*
IV World Conference on Lung Cancer, Secretariat Office, 342 MacLaren Street, Ottawa, Ontario, Canada.

**Twelfth L.H. Gray Conference: Assays of normal tissue injury, and their cellular intepretation**
*2–5 September 1985*    *Manchester, UK.*
Dr. J.H. Hendry, Paterson Laboratories, Christie Hospital & Holt Radium Institute, Manchester, M20 9BX, UK.

**European Nuclear Medicine Congress 1985**
*3–6 September 1985*    *London, UK*
European Nuclear Medicine Congress 1985, Institute of Nuclear Medicine, Middlesex Hospital Medical School, Mortimer Street, London W1N 8AA, UK.

**International Symposium on Ovarian Cancer**
*11–13 September 1985*    *Glasgow, UK*
Dr A. Belfield, Biochemistry Department, Royal Maternity Hospital, Glasgow G4 0NA, UK.

**Symposium: Radiation Carcinogenesis Molecular and Biological Aspects**
*23–25 September 1985*    *Liege, Belgium*
Dr Jacques Boniver, Secretary, Université de Liège Laboratoire d'Anatomie Pathologique, rue des Bonnes Villes, 1-B-4020 Liege, Belgium.

**Conference on Chemical Modifiers of Cancer Treatment**
*20–24 October 1985*    *Clearwater, Florida, USA*
Ms Suzanne Bohn, American College of Radiology, 925 Chestnut Street, Philadelphia, PA 19107, USA.

**Second International Symposium on Detection and Treatment of Minimal Residual Disease in Acute Leukemia**
*6–8 November 1985*    *Rotterdam, The Netherlands*
Dr J.W. van der Velden, Organizing Committee Leukemia Symposium, Dr Daniel den Hoed Cancer Centre, PO Box 5201, 3008 Rotterdam, The Netherlands.

**First International Congress on Neoadjuvant Chemotherapy**

*7–9 November 1985 Paris, France*

SOMPS – 1st International Congress on Neoadjuvant Chemotherapy, Hopital de la Salpétrière, 47 Boulevard de l'Hopital, 75651 Paris, France.

**Indian Society of Oncology: Second Biennial Conference**

*9–15 February 1986 Taj Mahal Hotel, Bombay, India*

Dr Ernest Borges Marg, Tata Memorial Hospital, Parel, Bombay 400 012, India.

**Fourteenth International Cancer Congress**

*21–27 August 1986 Budapest, Hungary*

Professor S. Eckhardt, National Institute of Oncology, Rath György u7/9, 1525 Budapest, Hungary.

**Fourth International Congress on Seriology**

*1–4 September 1986 Paris, France*

Dr. J. Gest, Centre Rene Hiquenin, 35 rue Dailly, 92211, Saint Cloud, France.